# Spatial Variation and Predictors of Women’s Sole Autonomy in Healthcare Decision-Making in Bangladesh: A Spatial and Multilevel Analysis

**DOI:** 10.3390/healthcare12242494

**Published:** 2024-12-10

**Authors:** Satyajit Kundu, Md Hafizur Rahman, Syed Sharaf Ahmed Chowdhury, John Elvis Hagan, Susmita Rani Dey, Rakhi Dey, Rita Karmoker, Azaz Bin Sharif, Faruk Ahmed

**Affiliations:** 1 School of Medicine and Dentistry, Griffith University, Gold Coast, QLD 4222, Australia; satyajitnfs@gmail.com (S.K.); f.ahmed@griffith.edu.au (F.A.); 2 International Centre for Diarrhoeal Disease Research, Bangladesh (ICDDR, B), Mohakhali, Dhaka 1212, Bangladesh; hafiz.rahman@icddrb.org; 3 Department of Public Health, North South University, Dhaka 1229, Bangladesh; sharaf.chowdhury@northsouth.edu (S.S.A.C.); azaz.sharif@northsouth.edu (A.B.S.); 4 Global Health Institute, North South University, Dhaka 1229, Bangladesh; 5 Department of Health, Physical Education and Recreation, College of Education Studies, University of Cape Coast, PMB TF0494, Cape Coast P.O. Box 5007, Ghana; 6 Neurocognition and Action-Biomechanics-Research Group, Faculty of Psychology and Sports Science, Bielefeld University, Postfach 10 01 31, 33501 Bielefeld, Germany; 7 Statistics Discipline, Khulna University, Khulna 9208, Bangladesh; susmitardey@gmail.com (S.R.D.); rakhidey.stat@gmail.com (R.D.); 8 Department of Management Studies, Bangabandhu Sheikh Mujibur Rahman Science and Technology University, Gopalganj 8100, Bangladesh; rkarmoker44@gmail.com

**Keywords:** healthcare autonomy, healthcare decision-making, spatial analysis, Bangladesh

## Abstract

**Background:** Knowing the spatial variation and predictors of women having sole autonomy over their healthcare decisions is crucial to design site-specific interventions. This study examined how women’s sole autonomy over their healthcare choices varies geographically and what factors influence this autonomy among Bangladeshi women of childbearing age. **Methods:** Data were obtained from the Bangladesh Demographic and Health Survey (BDHS) 2017–18. The final analysis included data from a total of 18,890 (weighted) women. Spatial distribution, hot spot analysis, ordinary Kriging interpolation, and multilevel multinomial regression analysis were employed. **Results:** The study found that approximately one in ten women (9.62%) exercised complete autonomy in making decisions about their healthcare. Spatial analysis revealed a significant clustering pattern in this autonomy (Moran’s I = 0.234, *p* < 0.001). Notably, three divisions—Barisal, Chittagong, and Sylhet—emerged as hot spots where women were more likely to have sole autonomy over their healthcare choices. In contrast, the cold spots (poor level of sole healthcare autonomy by women) were mainly identified in Mymensingh and Rangpur divisions. Women in the age group of 25–49 years, who were highly educated, Muslim, urban residents, and had not given birth recently were more likely to have sole autonomy in making healthcare decisions for themselves. Conversely, women whose husbands were highly educated and employed, as well as those who were pregnant, were less likely to have sole autonomy over their healthcare choices. **Conclusions:** Since the spatial distribution was clustered, public health interventions should be planned to target the cold spot areas of women’s sole healthcare autonomy. In addition, significant predictors contributing to women’s sole healthcare autonomy must be emphasized while developing interventions to improve women’s empowerment toward healthcare decision-making.

## 1. Introduction

Women’s sole autonomy in decision-making refers to their ability to independently make and implement their own choices at home, significantly impacting maternal and child healthcare and outcomes [1,2]. Women need to have control over their own choices to seek and acquire healthcare services for themselves and their children. However, when men hold more power in relationships, in certain societies, it stops women from making their own healthcare decisions, making it harder to receive the healthcare they need [3]. Husbands and wives, as couples, have a shared social connection that impacts their healthcare choices [4]. In addition, women from developing countries, especially South Asian women, are largely marginalized in the decision-making process [5]. Lack of healthcare autonomy among women may result in high rates of maternal and infant mortality, particularly in developing countries [2]. Poor healthcare autonomy also contributes to low levels of nutrition [6] and poor reproductive health in women [7]. It has been advocated that empowering women in healthcare decision-making positively impacts the health of mothers and their children [8].

Previous studies showed that the benefits of greater women’s healthcare autonomy include lower long-term fertility, better child survival rates, and resource distribution in the household’s best interests [9]. The utilization of maternal healthcare services in developing countries like Bangladesh is significantly influenced by women’s autonomy, as it has been reported previously [10]. Previous research exploring the relationships between women’s social standing, empowerment, and autonomous decision-making revealed that studies used varied approaches to quantify women’s independence in healthcare choices [11,12]. In Bangladesh, the level of women’s independence is frequently assessed by considering various factors such as their ability to make decisions, manage finances, and move freely depending on their ability to manage their material and financial resources and their freedom of movement within their community [10]. Women’s autonomy to make independent decisions is influenced by various societal and personal factors. These include societal norms, cultural practices, established gender roles, inequalities between sexes, religious beliefs, economic status of the household, urban or rural living environments, access to media, and various behavioral aspects [13,14,15]. Additionally, factors like economic status and poverty, religious beliefs, and employment opportunities could be linked to the levels of women’s autonomy in making healthcare decisions [16,17].

Literature suggests that there has been a rise in women’s autonomy in the use of maternal healthcare in developing countries over the past decade [2,13]. Women seldom have equal access to healthcare, education, and work as compared to their male counterparts worldwide, particularly in low- and middle-income nations [18]. Women’s health is sometimes viewed as being of secondary concern [19]. According to a study conducted in South Asian nations, the majority of women do not participate in making their own healthcare decisions in Nepal, and the same is true in almost half of families in Bangladesh and India [1]. Previous studies conducted in Ethiopia [2], Ghana [20], and Nepal [21] have shown that women’s autonomy increases with factors such as age, level of education, employment status, and the number of children they have, along with their partner’s education and employment status. A study conducted in Bangladesh reveals a link between decision-making, whether made alone or collaboratively with a spouse and the usage of maternal healthcare services. To encourage greater utilization of maternal health care, it emphasizes improved spousal collaboration and communication [15]. It is crucial to raise awareness of women’s advancements in education, work, and general household influence in decision-making in Bangladesh, where male domination in decision-making is still largely believed to be the norm [15].

The concept of women’s autonomy encompasses multiple facets, making it crucial to identify the key social elements and variables that significantly influence it [15]. While earlier research in Bangladesh has explored the prevalence and factors influencing women’s healthcare autonomy, these studies defined autonomy as including both independent decision-making and shared decision-making with spouses. This approach did not distinguish between sole and joint autonomy in healthcare choices [1,22,23]. However, it is reported that when a person can act on their own behalf, that is, to make decisions about their own personal issues, that person is considered independent [24]. As a result, women who make their own decisions independently about their healthcare should be considered autonomous [25]. Besides, the results of these studies fall short of capturing the spatial distribution of women’s sole autonomy in healthcare decision-making. A study conducted in Ethiopia revealed that women’s autonomy in healthcare decisions follows a non-random pattern. The study emphasized the importance of identifying hot spots of autonomy and mapping its geographical distribution across the country. This spatial understanding is vital for developing targeted initiatives and policies aimed at reducing gender-based inequalities in healthcare access [2].

Therefore, it is crucial to identify geographic regions where impoverished women have less autonomy over their healthcare decisions using Geographic Information Systems (GIS) to design site-specific interventions. We employed both hot spot analysis and projection mapping methods to gain a thorough spatial perspective on women’s healthcare autonomy. Hot spot analysis highlights regions of both high and low autonomy, while projection mapping using the interpolation technique enables us to display the value of a variable at one or more unknown locations, considering the known values near the areas of interest [26,27], providing complementary insights that enhance our capacity to reach policy-relevant conclusions. In addition, by identifying the factors contributing to women’s low participation in healthcare decision-making, nations can better establish policies and initiatives addressing gender disparities in healthcare [28]. Therefore, this study aimed to ascertain the spatial variation and factors influencing sole autonomy over healthcare decision-making among Bangladeshi women aged 15–49 years in Bangladesh.

## 2. Method

### 2.1. Study Design and Sampling

The study utilized data from the Bangladesh Demography and Health Survey (BDHS) 2017–18, a nationally representative survey employing a two-stage stratified cluster sampling approach. In the initial stage, 675 enumeration areas (EAs) were chosen using probability proportional to size sampling. The second stage involved selecting 30 households from each EA through systematic random sampling. These EAs served as the primary sampling units (PSUs) for the survey. In BDHS 2017–18, a total of 20,250 households were selected with 20,376 eligible women aged 15 to 49 years to be interviewed. Eventually, data from 20,127 Bangladeshi women were collected in BDHS 2017–18. The details of the sampling technique are briefly discussed elsewhere [29]. In this study, while managing the dataset, we excluded the missing cases from the outcome and all explanatory variables. After excluding the missing cases, we included data from a weighted sample of 18,890 married reproductive-aged (15–49 years) women in the final analysis of this study.

### 2.2. Outcome Variable

This study measured the sole autonomy for healthcare decision-making as the outcome variable. The autonomy in healthcare decision-making is measured by DHS based on who usually makes decisions regarding the healthcare of the respondent. Participating women were given four choices for answering the question. Women could have responded as making their own healthcare decisions, making a joint decision with their partner, only the partner making decisions about their healthcare, or other family members making this decision for her [29]. For the sake of our analysis, the responses regarding healthcare autonomy were recoded as (1) woman alone, (2) jointly with husband, and (3) others. Women who made the decision independently were considered as having sole healthcare decision-making autonomy; when the decision was taken solely by partners or other family members, it was categorized as ‘decided by others’ [4,25].

### 2.3. Explanatory Variables

Based on a review of existing research, this study selected a set of explanatory variables for analysis [1,2,4,20,25,30,31]. The factors considered in the investigation included: age of women (15–24 years, 25–34 years, 35–49 years), educational status of women and their husbands (no education, primary, secondary, higher), employment status of the women (working, not working), husband’s occupation (does not work, services/job, business, agriculture, others), religion (Muslim, others), parity (none, 1–2, and 3 or more), place of residence (urban, rural), and administrative division. Exposure to the media included the ability to access and/or read newspapers/magazines, watch television, and listen to radio, which were combined and categorized as having been exposed to media or not having been exposed to media. In addition to the responses for the variables, current pregnancy and birth in the last 3 years (yes, no) were also considered. To determine household wealth status, a principal component analysis (PCA) was conducted, evaluating the possession of various household assets [29]. The resulting wealth index was then divided into five categories: poorest, poorer, middle, richer, and richest quintiles.

### 2.4. Statistical Analysis

The analysis of data was conducted using two software packages: STATA version 17.0, developed by StataCorp in College Station, Texas, and ArcGIS version 10.8. The weighted prevalence of sole decision-making autonomy for healthcare was calculated by considering respective weights for the unit of analysis. We also incorporated a complex survey design using “*svyset*” in STATA to adjust for cluster effects and sample stratification, and the “*svy*” command was consequently used to obtain valid statistical inferences. Given that the BDHS 2017–18 employed a two-stage stratified cluster sampling method, resulting in a hierarchical data structure, a multilevel regression model was deemed the most suitable analytical approach. This technique was chosen to effectively account for and analyze any variations specific to the clusters in the sample [32]. The study employed multilevel multinomial regression modeling to analyze the factors influencing women’s sole autonomy in healthcare decisions. This approach was chosen because the outcome variable consisted of three categories. In the analysis, clusters were treated as level-2 factors to account for the hierarchical data structure. Two separate models (Model 1 and Model 2) were developed to compare women’s sole decision-making autonomy against two alternatives: decisions made jointly with husbands and decisions made by others.

In Model 1, “decision-making by others” was considered as the base category to identify the predictors of sole autonomy in healthcare decision-making among women in comparison with “joint decision-making”. In Model 2, “joint decision-making” was considered as the base category to find out the predictors of sole autonomy in healthcare decision-making among women while being compared to “decision-making by others”. Besides, for multilevel modeling, we applied an intercept-only model (null model) first without taking any predictor keeping clusters as a level 2 factor to estimate the cluster-level variance in the outcome variable. The null model indicated a significant cluster-level variance for both Model 1 (variance: 0.54, SE: 0.06) and Model 2 (variance: 0.45, SE: 0.05), which also highlights the appropriateness of applying a multilevel modelling approach. A generalized structural equation modeling technique using the “*gsem*” command in STATA was employed to conduct the multilevel regression analysis. The analysis estimated both fixed effects of explanatory variables and random effects at the cluster level. Before running the regression models, multicollinearity was assessed using variance inflation factors. Results were interpreted using adjusted relative risk ratios (aRRR) with 95% confidence intervals, considering *p*-values below 0.05 as statistically significant.

### 2.5. Spatial Analysis and Autocorrelation

The weighted prevalence of sole healthcare decision-making autonomy was calculated for each cluster using STATA. This cluster-wise prevalence was then merged with the cluster number and geographic coordinate data (point shapefile) of each cluster using ArcGIS software. For the BDHS 2017–18, global positioning system (GPS) data of 672 clusters were found among 675 clusters included in the survey. Three of these clusters did not have any location data and the final study identified data from a total of 672 clusters. For spatial analysis, the refined dataset was transferred to Excel before being imported into ArcGIS 10.8 software. To examine the geographical distribution of women’s independent healthcare decision-making in Bangladesh, the study employed the spatial autocorrelation technique, specifically the Global Moran’s I statistic. This metric helps determine whether the pattern of healthcare autonomy is clustered, dispersed, or random across the country. A Moran’s I value approaching +1 suggests that healthcare autonomy is concentrated in specific regions. Conversely, a value near −1 indicates a scattered pattern, while a value close to 0 implies a random distribution of healthcare autonomy throughout the area [33].

### 2.6. Hot Spot Analysis

Gettis-OrdGi* statistics were determined to identify the hotspots for assessing the statistical significance of the clustering of sole autonomy in healthcare decision-making across clusters at various degrees of significance. The analysis of the hotspot calculates the Z-score and determines the *p*-value [34]. The *p*-value used in this study determined if there was substantial clustering at a 99%, 95%, or 90% confidence level, respectively. Areas with a greater prevalence of sole decision-making autonomy for healthcare (hotspots) and those with a lower prevalence (cold spots) were identified using statistical outputs with high Gi* and low Gi*, respectively [35].

### 2.7. Spatial Interpolation

To understand the state of sole autonomy in healthcare decision-making in Bangladesh, it is highly expensive and time-consuming to gather trustworthy data throughout the whole nation. One method of projecting this outcome (sole healthcare autonomy in Bangladesh) is to use sampled clusters to forecast regions that were not sampled. Based on the EAs from which a sample was obtained, the Ordinary Kriging spatial interpolation approach was used to forecast sole autonomy in healthcare decision-making in regions where data were not obtained by the BDHS 2017. The ordinary Kriging interpolation was employed with a spherical semivariogram model and a 12-point variable radius extent, while conversion of units was used as the cell size projection method to interpolate the point values.

## 3. Results

### 3.1. Background Characteristics of the Participants

Table 1 contains the background characteristics of the study participants. A total of 18,890 respondents aged between 15 to 49 years were included, where 9.62% of respondents had sole healthcare autonomy, 66.84% of women decided jointly with their husbands, and 23.54% of women’s healthcare was decided by others. Among the respondents, 15.55% received no education, and 52.93% of women were reported to be unemployed. The distribution of respondents was nearly even across the wealth index, which covered the spectrum from the poorest (18.33%) to the richest (20.83%), where the prevalence of sole autonomy in healthcare increased by wealth status, from 7.62% in women from poorest households to 10.63% in women from richest families. The majority of women were Muslim, with a higher prevalence (9.96%) of sole autonomy in healthcare compared to 6.32% in non-Muslims. A greater proportion of the women resided in rural areas (71.66%), while the proportion of women having sole autonomy in healthcare was higher in urban areas (10.68%) than in rural areas (9.20%). The highest number of participants were from Dhaka (25.62%) followed by Chittagong (17.88%).

### 3.2. Spatial Distribution and Hot Spot Analysis

**Figure 1:**
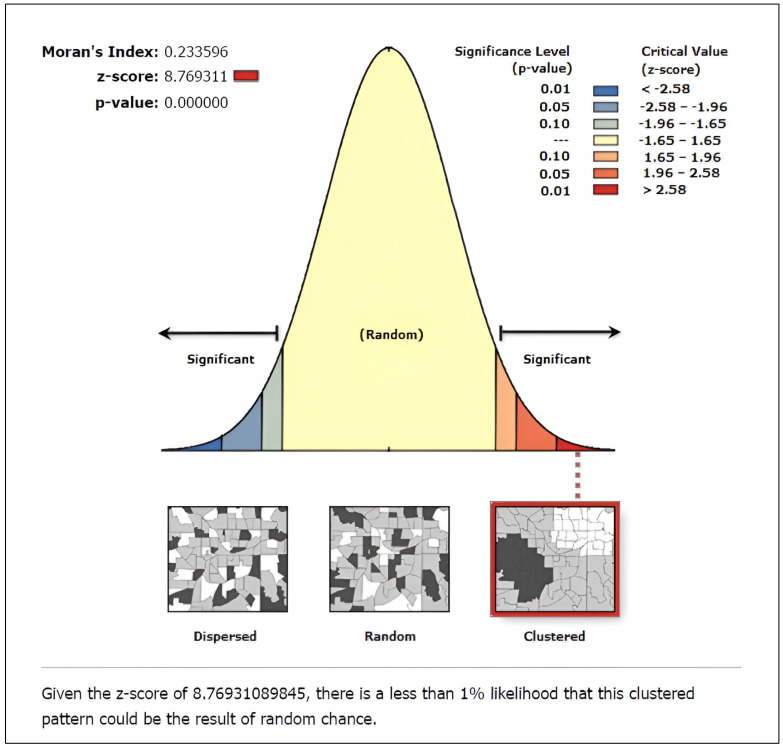
Global spatial autocorrelation report showing the women’s sole decision-making autonomy in healthcare in Bangladesh (map was generated using ArcGIS v 10.8 software).

A map was created to illustrate the spatial distribution of women’s autonomy in healthcare decision-making (Supplementary Figure S1). The global spatial autocorrelation analysis revealed that the distribution of women’s sole healthcare autonomy across Bangladesh was not random. Instead, it showed a clustered pattern (global Moran’s I = 0.234, *p* < 0.001) (Figure 1).

**Figure 2:**
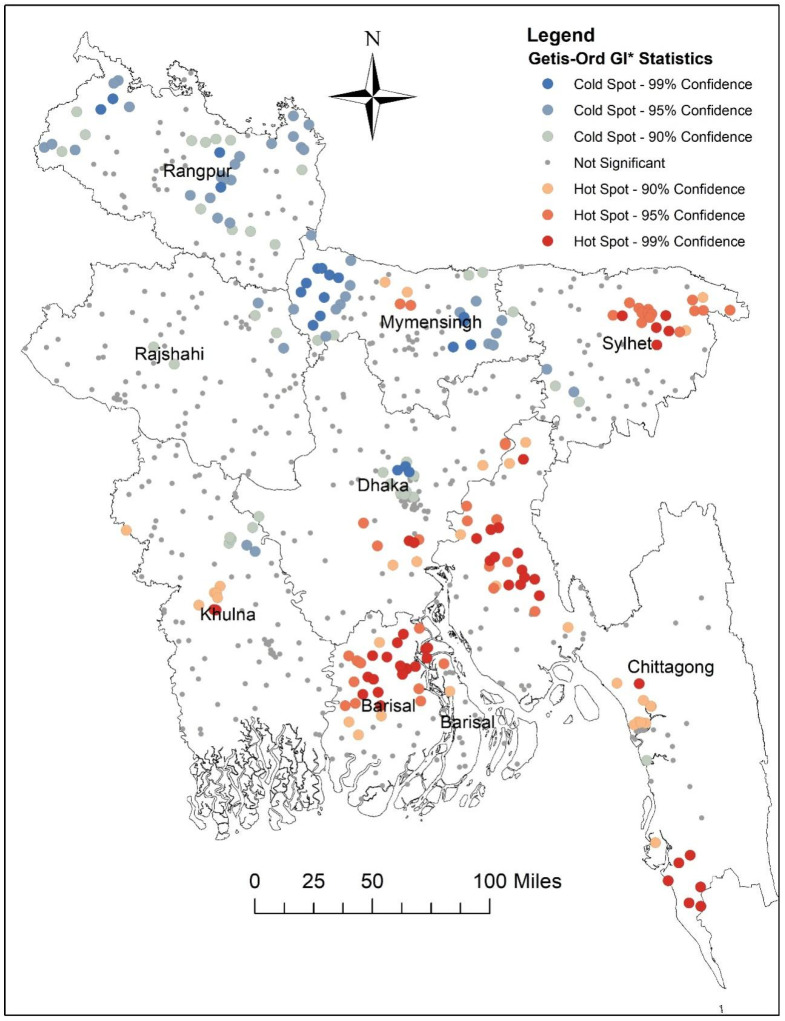
Spatial clustering (hot spot and cold spot) of women’s sole decision-making autonomy in healthcare in Bangladesh (map was generated using ArcGIS v 10.8 software).

The hot spots for higher prevalence of women’s sole healthcare autonomy were identified using red color and the cold spots with blue color, as shown in the map. Hot spot areas (high prevalence of women’s sole healthcare autonomy) were located in Barisal, Chittagong, and Sylhet divisions, whereas the cold spots (lower prevalence of women’s sole healthcare autonomy) were mainly identified in Mymensingh and Rangpur divisions (Figure 2).

### 3.3. Ordinary Kriging Interpolation Analysis

**Figure 3:**
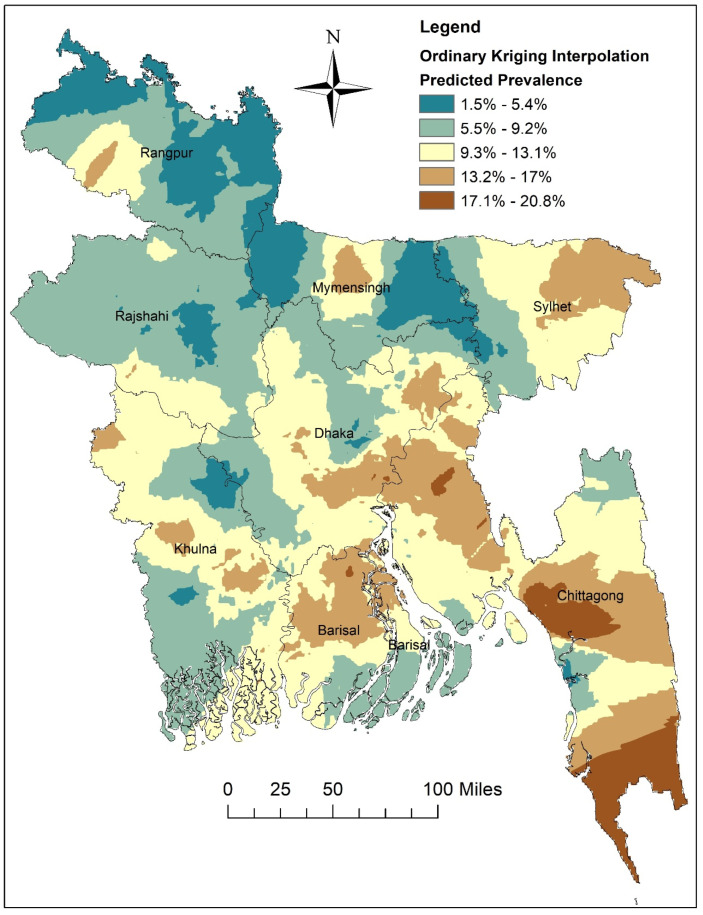
Spatial interpolation of women’s sole autonomy in healthcare decision-making in Bangladesh (map was generated using ArcGIS v 10.8 software).

The highest predicted prevalence of women’s sole autonomy in healthcare decision-making was observed in the southern part of the Chittagong division. In addition, the moderate level predicted prevalence of women’s sole autonomy in healthcare decision-making, ranging from 13.2% to 17%, was identified in Barisal, Chittagong, Sylhet, and the eastern part of Dhaka division. In contrast, the interpolation shows that the lowest predicted prevalence was determined in Mymensingh, Rangpur, and small parts of the Rajshahi and Khulna divisions (Figure 3).

### 3.4. Predictors of Women’s Sole Autonomy in Healthcare Decision-Making

Table 2 shows the multilevel multinomial regression analysis to determine the likelihood of women having sole autonomy in healthcare decision-making compared with women making joint decisions with their husbands and decisions being made by others. Table 2 also demonstrates the random effects (measured as variance) at the cluster level, which indicated that the likelihood of women having sole healthcare autonomy from different clusters varied significantly (variance: 0.39, SE: 0.05 for Model 1, and variance: 0.37, SE: 0.05 for Model 2).

This section discusses women’s healthcare autonomy in relation to individuals who made decisions about their healthcare solely and those who made decisions together with their husbands. Women’s sole healthcare autonomy was higher among those from 25–34 years age group (aRRR: 1.39, 95% CI: 1.18–1.64) and 35–49 years age group (aRRR: 1.64, 95% CI: 1.34–2.00) compared to those from 15–24 years age group. The risk of women’s sole healthcare autonomy was reduced with the education level of their husbands. Similarly, individuals having working husbands were less likely to have sole autonomy over healthcare decision-making. Muslim women had a higher risk of sole healthcare decision-making autonomy compared to those who follow other religions (aRRR: 1.66, 95% CI: 1.33–2.07). Currently, pregnant women (aRRR: 0.52, 95% CI: 0.39–0.71) were associated with a lower risk of having sole healthcare autonomy compared to those who were not pregnant. Not giving birth in the last three years was associated with a higher risk of having sole healthcare autonomy (aRRR: 1.17, 95% CI: 1.01–1.35). Women hailing from the most impoverished households exhibited lower levels of healthcare autonomy in comparison to those from the most affluent households (aRRR: 0.78, 95% CI: 0.62–0.99) (Table 2).

In this section, the predictors of women’s sole autonomy in healthcare decision-making were determined in comparison with women whose healthcare decisions were taken by others. The women’s sole healthcare autonomy was higher among those from 25–34 years age group (aRRR: 2.26, 95% CI: 1.89–2.70) and 35–49 years age group (aRRR: 2.94, 95% CI: 2.36–3.65) compared to those from 15–24 years age group. The risk of women having sole healthcare autonomy was increased with the education level of women, while the risk was reduced with the education level of their husbands. Working women showed a higher risk of having sole healthcare autonomy compared to those who are not working (aRRR: 1.34, 95% CI: 1.18–1.52). However, individuals with working husbands were less likely to have sole autonomy over healthcare decision-making compared to those having husbands who do not work. Muslim women had a higher risk of sole healthcare autonomy compared to those who follow other religions (aRRR: 1.71, 95% CI: 1.35–2.17). Pregnant women (aRRR: 0.60, 95% CI: 0.44–0.82) were associated with a lower risk of having sole healthcare autonomy compared to those who were not pregnant. Women having parity of 1 to 2 (aRRR: 1.64, 95% CI: 1.30–2.07) and 3 or more (aRRR: 1.53, 95% CI: 1.17–2.01) were associated with a higher risk of having sole healthcare autonomy compared to those having no children. Similarly, not giving birth in the last three years was associated with a higher risk of having sole healthcare autonomy (aRRR: 1.35, 95% CI: 1.15–1.58) compared to those who did not give birth. Women from urban areas had 1.32 times higher likelihood of having sole healthcare autonomy compared to rural women (aRRR: 1.32, 95% CI: 1.11–1.57) (Table 2).

## 4. Discussion

This research offers valuable insights into women’s sole healthcare decision-making autonomy in Bangladesh, elucidating the multifaceted influences of demographic, socioeconomic, and spatial factors. The findings reveal a notable deficit in the sole autonomy of women over their healthcare decisions, with only a minority (9.62% of respondents) exercising independent healthcare decision-making, while the majority either engage in joint decision-making with their spouses (66.84%) or have their healthcare choices determined by others (23.54%). This proportion is considerably lower compared to the pooled prevalence reported in a review study focused on low- and middle-income countries (LMICs), which found that, on average, 55.15% of women had the autonomy to make decisions regarding their maternal healthcare services [36]. Additionally, a separate study focusing on married young women in Bangladesh found that approximately one-third (31%) exhibited a higher autonomy over healthcare decision-making [37]. This is consistent with findings from previous research in South Asian countries, where a similar trend was noted in women’s participation in healthcare decision-making, with 72.7% in Nepal, 54.3% in Bangladesh, and 48.5% in India [1].

The disparities in findings may stem from various contextual factors, though one study conducted in Bangladesh suggests that enhanced cooperation between spouses on household and health matters could lead to increased utilization of maternal health services [1]. The consistently low rates of women’s independence in healthcare decisions are concerning, as research has shown this lack of autonomy contributes to underutilization of maternal health services in countries like Nepal [38] and India [39]. A qualitative study in Bangladesh uncovered a significant communication gap between husbands and wives regarding sexual and reproductive health matters. This lack of dialogue makes it challenging for men to understand and respond to their partners’ reproductive health concerns. [40]. However, given Bangladesh’s cultural context, where men traditionally hold decision-making power within families, excluding them from discussions and decisions about maternal health could actually hinder their capacity to make well-informed choices for their wives’ or partners’ wellbeing.

Spatial analysis was employed to examine the geographic distribution of women’s healthcare autonomy, revealing clustering patterns across different regions of Bangladesh (global Moran’s I = 0.234, *p* < 0.001). Hotspot analysis identified areas with a higher prevalence of sole healthcare autonomy, predominantly observed in the Barisal, Chittagong, and Sylhet divisions, while a lower prevalence of sole autonomy was evident in the Mymensingh and Rangpur divisions, suggesting a need for increased resource allocation in these regions. Notably, the Mymensingh and Rangpur divisions, situated in the extreme northern part of the country and sharing borders with India, exhibit distinctive socio-economic characteristics that contribute to their comparatively lower autonomy rates. The Rangpur division continues to experience the highest concentration of poverty, with limited infrastructural development and poor industrialization contributing to persistent poverty rates. With over 44% of households in the poorest quintile, this historically impoverished northern region lags in terms of wealth compared to other divisions [41]. Similarly, the Mymensingh division, with 39.57% of households in the poorest quintile, exhibits socio-economic indicators reflective of economic challenges [41]. Hence, the economic challenges faced by residents of Rangpur and Mymensingh divisions likely play a significant role in the diminished rates of women’s decision-making within these regions.

Moreover, both Rangpur and Mymensingh divisions report lower female literacy rates, with 68% and 65%, respectively, compared to the national average of 73% [42]. This discrepancy in educational attainment among women may further contribute to the observed low rates of women’s healthcare autonomy in these regions, as indicated in our analysis. We found that women with higher levels of education demonstrate a higher propensity for sole decision-making, consistent with evidence from systematic reviews in LMICs [36] and studies in Nepal [21], Ethiopia [2], and Sub-Saharan Africa [43]. Education appears to empower women to assume greater control over healthcare decisions. Through education, women gain access to valuable information about health, reproductive rights, and healthcare services, enabling them to make informed choices about their own well-being.

As women age, there is a notable increase in the likelihood of both sole decision-making and joint decision-making with spouses or others regarding healthcare, which is consistent with findings from a systematic review conducted in LMICs [36]. These findings suggest that advancing age may foster greater confidence or assertiveness in healthcare decision-making, possibly influenced by a desire for greater autonomy and mutual respect within marital relationships. However, it is noteworthy that the influence of a mother-in-law’s authority persists in Bangladesh, despite increasing autonomy with age [30].

Additionally, employed women are more likely to engage in joint decision-making, a pattern also observed in South Asia [1] and Ethiopia [2], likely due to increased financial independence and bargaining power associated with employment. Furthermore, urban residence is associated with a higher likelihood of sole decision-making autonomy for healthcare in our study, as supported by findings from meta-analyses in LMICs [36] and developing countries [39] as well as South Asia [1]. This may be attributed to urban women’s access to diverse informational resources, including reproductive rights and policy information disseminated through various media channels.

The occupation of husbands also plays a crucial role in shaping women’s decision-making autonomy in healthcare matters. Husbands’ involvement in income-generating activities negatively impacts women’s decision-making autonomy, highlighting the dynamics of economic influence within households. One study in Bangladesh reported that men often perceive women’s involvement in development initiatives as a threat to their own dominance and authority within society [44].

Moreover, being Muslim is positively associated with sole decision-making autonomy for women in healthcare matters, indicating the influence of cultural norms and religious beliefs on decision-making power distribution within households. Interestingly, pregnancy status negatively affects women’s autonomy in healthcare decision-making, underscoring the importance of considering reproductive status when assessing women’s agency in healthcare decision-making processes.

A key strength of the study is its use of nationally representative data from the BDHS 2017–18, which offer a thorough insight into women’s autonomy in healthcare decision-making throughout the country. Additionally, the study’s incorporation of geospatial analysis enables the identification of areas where resources for promoting women’s autonomy in healthcare decision-making are most needed, facilitating targeted interventions and policy formulation to address regional disparities effectively. The study’s limitations include its dependence on self-reported data, which could be influenced by recall bias or social desirability bias, potentially influencing responses regarding healthcare decision-making autonomy. Additionally, while the study accounts for various socio-demographic factors, other important determinants such as cultural norms, marital dynamics, and access to healthcare services may not have been fully captured. Also, due to the lack of information in the dataset, the measurement of sole autonomy over healthcare was based on a single item, which may not cover the aspects of knowledge, attitude towards spousal relationships, social support, healthcare accessibility, and cultural issues that may significantly influence women’s autonomy in healthcare. Besides, using a single question may not fully capture the nuanced nature of how sole vs shared decision-making reflects the ethical concept of autonomy among women, and further qualitative studies could be designed for an in-depth exploration of this issue. The cross-sectional nature of the study restricts the ability to determine cause-and-effect relationships between the factors examined and women’s independence in making healthcare decisions. As the DHS program provides geographic coordinates only at the cluster level, not for individual households, it was not possible to perform interpolation based on individual households. Lastly, although the study employs advanced statistical techniques, the complexity of the models utilized may present challenges in interpretation and may require further validation through replication studies or qualitative research to corroborate findings and enhance their robustness.

## 5. Conclusions

The findings underscore a concerning deficit in women’s sole healthcare decision-making autonomy, with most respondents either engaging in joint decision-making with their spouses or having their healthcare choices determined by others. Rangpur and Mymensingh divisions have the lowest rates of women’s sole healthcare decision-making autonomy. Furthermore, the autonomy of women in making healthcare decisions is influenced by various factors, such as the age of women, the education level of both women and their husbands, employment status, husband’s occupation, religious beliefs, number of children, place of living, administrative area, exposure to media, current pregnancy status, previous childbirth experiences, and household wealth. To address the observed disparities in women’s autonomy in healthcare decision-making in Bangladesh, targeted interventions are essential. Initiatives should prioritize educational programs to enhance women’s literacy and knowledge about healthcare rights. Economic empowerment efforts, such as skill development and job opportunities, could also be implemented to bolster women’s financial independence.

## Figures and Tables

**Table 1:** Distribution of survey participants based on background characteristics, and who decides on women’s healthcare in Bangladesh (n = 18,890).

Variables	Weighted Total, n (%)	Percentage by Who Decides on Women’s Healthcare		
		Woman Alone; % (SE)	Jointly with Husband; % (SE)	Decided by Others; % (SE)
**Overall prevalence (95% CI)**		9.62 (9.21–10.05)	66.84 (66.17–67.51)	23.54 (22.94–24.15)
**Age of women**				
15–24 years	5402 (28.59)	6.47 (0.33)	59.93 (0.67)	33.60 (0.64)
25–34 years	6723 (35.59)	10.11 (0.37)	69.91 (0.56)	19.98 (0.49)
35–49 years	6766 (35.82)	11.66 (0.39)	69.31 (0.56)	19.04 (0.48)
**Education of women**				
No education	2937 (15.55)	10.30 (0.56)	67.64 (0.86)	22.06 (0.77)
Primary	5873 (31.09)	10.32 (0.40)	67.30 (0.61)	22.38 (0.54)
Secondary	7636 (40.42)	9.19 (0.33)	65.57 (0.54)	25.24 (0.50)
Higher	2444 (12.94)	8.48 (0.56)	68.73 (0.94)	22.79 (0.85)
**Women employment status**				
Working	8892 (47.07)	9.13 (0.31)	70.93 (0.48)	19.94 (0.42)
Not working	9998 (52.93)	10.06 (0.30)	63.20 (0.48)	26.74 (0.44)
**Husbands’ education**				
No education	4071 (21.55)	11.60 (0.50)	67.11 (0.74)	21.29 (0.64)
Primary	6070 (32.13)	8.56 (0.36)	67.83 (0.60)	23.61 (0.55)
Secondary	5656 (29.94)	9.71 (0.39)	64.54 (0.64)	25.75 (0.58)
Higher	3093 (16.38)	8.95 (0.51)	68.73 (0.83)	22.32 (0.75)
**Husbands’ occupation**				
Don’t work	401 (2.13)	22.95 (2.10)	53.09 (2.49)	23.97 (2.13)
Services/job	3718 (19.68)	10.89 (0.51)	67.55 (0.77)	21.56 (0.67)
Business	3407 (18.03)	7.99 (0.46)	66.30 (0.81)	25.71 (0.75)
Agriculture	4841 (25.63)	6.06 (0.34)	70.20 (0.66)	23.74 (0.61)
Others	6523 (34.53)	11.58 (0.40)	65.07 (0.59)	23.35 (0.52)
**Religion**				
Muslim	17,114 (90.60)	9.96 (0.23)	66.40 (0.36)	23.64 (0.32)
Others ^#^	1776 (9.40)	6.32 (0.58)	71.08 (1.08)	22.60 (0.99)
**Exposure to media**				
Yes	12,499 (66.17)	9.95 (0.27)	67.14 (0.42)	22.91 (0.38)
No	6391 (33.83)	8.97 (0.36)	66.26 (0.59)	24.77 (0.54)
**Currently pregnant**				
Yes	1131 (5.99)	5.51 (0.68)	64.68 (1.42)	29.81 (1.36)
No	17,759 (94.01)	9.88 (0.22)	66.98 (0.35)	23.14 (0.32)
**Parity**				
None	1965 (10.40)	7.25 (0.59)	54.35 (1.12)	38.39 (1.10)
1–2	10,138 (53.67)	9.17 (0.29)	68.05 (0.46)	22.78 (0.42)
3 or more	6787 (35.93)	10.99 (0.38)	68.65 (0.56)	20.36 (0.49)
**Birth in the last 3 years**				
No birth	13,931 (73.75)	10.43 (0.26)	67.35 (0.40)	22.22 (0.35)
Gave birth	4959 (26.25)	7.35 (0.37)	65.41 (0.68)	27.24 (0.63)
**Household wealth status**				
Poorest	3463 (18.33)	7.62 (0.45)	69.74 (0.78)	22.65 (0.71)
Poorer	3716 (19.67)	7.89 (0.44)	67.25 (0.77)	24.86 (0.71)
Middle	3828 (20.27)	10.10 (0.49)	64.36 (0.77)	25.55 (0.70)
Richer	3948 (20.90)	11.54 (0.51)	64.96 (0.76)	23.50 (0.67)
Richest	3935 (20.83)	10.63 (0.49)	68.21 (0.74)	21.16 (0.65)
**Place of residence**				
Rural	13,537 (71.66)	9.20 (0.25)	65.68 (0.41)	25.12 (0.37)
Urban	5353 (28.34)	10.68 (0.42)	69.77 (0.63)	19.55 (0.54)
**Administrative division**				
Barisal	1052 (5.57)	12.21 (1.01)	60.23 (1.51)	27.56 (1.38)
Chittagong	3378 (17.88)	13.33 (0.58)	62.28 (0.83)	24.40 (0.74)
Dhaka	4840 (25.62)	10.21 (0.44)	68.83 (0.67)	20.96 (0.58)
Khulna	2201 (11.65)	9.18 (0.62)	67.46 (1.00)	23.36 (0.90)
Mymensingh	1464 (7.75)	6.59 (0.65)	74.03 (1.15)	19.38 (1.03)
Rajshahi	2635 (13.95)	7.57 (0.52)	69.38 (0.90)	23.05 (0.82)
Rangpur	2240 (11.86)	6.10 (0.51)	69.69 (0.97)	24.20 (0.90)
Sylhet	1080 (5.72)	10.17 (0.92)	55.51 (1.51)	34.32 (1.44)

SE: Standard Error, CI: Confidence Interval, ^#^ Others included Hindus, Christians, and Buddhists.

**Table 2:** Multinomial regression analysis showing the determinants of women’s sole decision-making autonomy for healthcare in Bangladesh.

Variables	Sole Decision-Making Autonomy for Healthcare			
	Model 1: Decision-Making by Woman Alone vs. Joint Decision-Making		Model 2: Decision-Making by Woman Alone vs. Decision-Making by Others	
**Fixed-effect results**	aRRR	95% CI	aRRR	95% CI
**Age of women**				
15–24 years (RC)				
25–34 years	1.39 ***	1.18–1.64	2.26 ***	1.89–2.70
35–49 years	1.64 ***	1.34–2.00	2.94 ***	2.36–3.65
**Education of women**				
No education (RC)				
Primary	1.08	0.91–1.28	1.27 *	1.05–1.53
Secondary	1.03	0.85–1.25	1.35 **	1.09–1.67
Higher	0.97	0.74–1.26	1.62 **	1.21–2.18
**Women employment status**				
Working	0.97	0.86–1.09	1.34 ***	1.18–1.52
Not working (RC)				
**Husbands’ education**				
No education (RC)				
Primary	0.69 ***	0.59–0.80	0.70 ***	0.59–0.82
Secondary	0.77 **	0.65–0.91	0.71 ***	0.59–0.85
Higher	0.63 ***	0.50–0.79	0.63 ***	0.49–0.80
**Husbands’ occupation**				
Don’t work (RC)				
Services/job	0.49 ***	0.37–0.65	0.69 *	0.49–0.96
Business	0.35 ***	0.26–0.47	0.40 ***	0.28–0.56
Agriculture	0.24 ***	0.18–0.32	0.30 ***	0.22–0.43
Others	0.50 ***	0.38–0.67	0.68 *	0.50–0.95
**Religion**				
Muslim	1.66 ***	1.33–2.07	1.71 ***	1.35–2.17
Others ^#^ (RC)				
**Exposure to media**				
Yes	0.97	0.85–1.11	1.06	0.91–1.22
No (RC)				
**Currently pregnant**				
Yes	0.52 ***	0.39–0.71	0.60 **	0.44–0.82
No (RC)				
**Parity**				
None (RC)				
1–2	0.84	0.67–1.04	1.64 ***	1.30–2.07
3 or more	0.76 *	0.60–0.98	1.53 **	1.17–2.01
**Birth in the last 3 years**				
No birth	1.17 *	1.01–1.35	1.35 ***	1.15–1.58
Gave birth (RC)				
**Household wealth status**				
Poorest	0.78 *	0.62–0.99	0.98	0.75–1.28
Poorer	0.83	0.67–1.03	0.92	0.73–1.17
Middle	1.02	0.84–1.23	1.00	0.81–1.24
Richer	1.11	0.93–1.30	1.06	0.88–1.28
Richest (RC)				
**Place of residence**				
Rural (RC)				
Urban	0.89	0.76–1.05	1.32 **	1.11–1.57
**Administrative division**				
Barisal (RC)				
Chittagong	0.91	0.69–1.20	1.18	0.88–1.57
Dhaka	0.58 ***	0.43–0.77	0.96	0.71–1.29
Khulna	0.65 **	0.48–0.86	0.90	0.66–1.22
Mymensingh	0.42 ***	0.31–0.57	0.85	0.61–1.18
Rajshahi	0.49 ***	0.36–0.66	0.70 *	0.51–0.95
Rangpur	0.45 ***	0.33–0.61	0.58 **	0.42–0.79
Sylhet	0.93	0.69–1.25	0.83	0.61–1.13
**Random-effect results**				
Variance (SE)	0.39 (0.05)	0.03–0.05	0.37 (0.05)	0.27–0.49

* *p* < 0.005, ** *p* < 0.01, *** *p* < 0.001, CI: Confidence Interval, aRRR: adjusted Relative Risk Ratio, RC: Reference Category, SE: Standard Error, ^#^ Others included Hindus, Christians, and Buddhists. Model 1: Predictors of sole decision-making autonomy were determined in comparison to decision-making jointly with husband. Model 2: Predictors of sole decision-making autonomy was determined in comparison to decision-making by others.

## Data Availability

The study used the Bangladesh Demographic and Health Survey 2017–18 data. The data sets are available at: https://dhsprogram.com/data/available-datasets.cfm (accessed on 7 June 2023).

## Supplementary Materials

The following supporting information can be downloaded at: https://www.mdpi.com/article/10.3390/healthcare12242494/s1, Figure S1: Spatial distribution of women’s sole autonomy in healthcare decision-making in Bangladesh based on sampling clusters.
