# Supplementary material for: Spatial Variation and Predictors of Women’s Sole Autonomy in Healthcare Decision-Making in Bangladesh: A Spatial and Multilevel Analysis
Source: Healthcare (Basel). 2024 Dec 10;12(24):2494. doi: 10.3390/healthcare12242494 (PMC11728150; doi:10.3390/healthcare12242494)
Supplement: Supplementary file 1 [file healthcare-12-02494-s001.zip › healthcare-3254869-supplementary.pdf]

Supplementary Material:

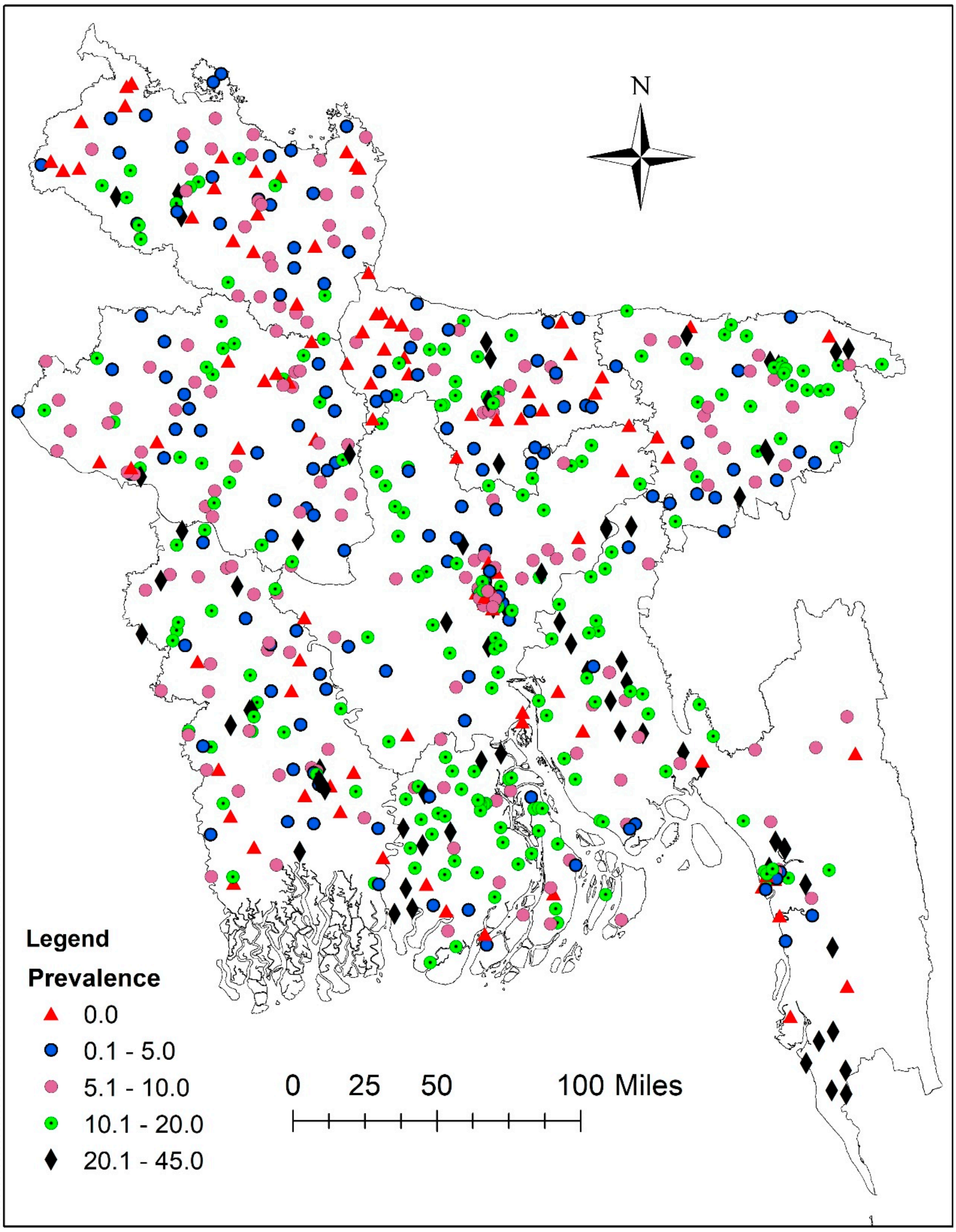

Suppl Figure S1. Spatial distribution of women's sole autonomy in healthcare decision-making in Bangladesh based on sampling clusters (map was generated using ArcGIS v 10.8 software)
